# Topical Egg yolk Oil and post-Laser recovery in atrophic acne scars: a retrospective propensity score-matched study with machine learning and SHAP analysis

**DOI:** 10.3389/fmed.2026.1932961

**Published:** 2026-09-25

**Authors:** Tingting Zhao, Dandan Li, Qin Huang, Xinhui Liu, Wenfeng Yang

**Affiliations:** Department of Dermatology, Affiliated Hospital of Panzhihua University, Panzhihua, China

**Keywords:** atrophic acne scars, egg yolk oil, fractional CO_2_ laser, machine learning, SHAP analysis

## Abstract

**Background:**

Prolonged epidermal recovery frequently complicates fractional carbon dioxide (CO_2_) laser therapy for atrophic acne scars. This study aims to evaluate the potential clinical utility of topical egg yolk oil (EYO) in post-laser wound management using machine learning and interpretability analyses.

**Methods:**

A propensity score-matched retrospective cohort of 324 patients was established. Clinical metrics, including wound healing time and ECCA scar scores, were extracted. Four machine learning models were developed to predict optimal clinical responses (ECCA reduction >= 30%), followed by SHapley Additive exPlanations (SHAP) analysis to explore non-linear feature associations.

**Results:**

The EYO group exhibited accelerated wound healing (5.5 vs. 8.5 days, *P* < 0.001) and superior 2-month ECCA reduction rates compared to the control group. The Random Forest model demonstrated robust predictive performance with an AUC of 0.889. SHAP analysis suggested a potential non-linear association, indicating that prolonged healing times might negatively correlate with optimal scar remodeling, while EYO intervention appeared to modulate the adverse effects of higher laser energy.

**Conclusion:**

Topical EYO may accelerate early epidermal repair and optimize medium-term aesthetic outcomes in laser-treated acne scars.

## 1 Introduction

Atrophic acne scars are common dermatological problems that may be accompanied by psychological conditions such as depression (1, 2). Currently, fractional carbon dioxide (CO₂) therapy is a popular technique that stimulates dermal remodeling to improve the appearance of these scars. Although effective, all ablative procedures may result in thermal injury, which can cause prolonged recovery in the epidermis (3, 4). Patients may experience acute post-op discomfort, residual erythema and post-inflammatory hyperpigmentation (5). Therefore, it is essential to find suitable skincare strategies after surgery in order to help the healing process and reduce any unwanted responses.

Post-laser wound care is largely devoted to the provision of a wound environment conducive to re-epithelialization, which is a moist and lipid-rich environment (6). A traditional topical agent called egg yolk oil is rich in lipids, proteins and vitamins (7). Recent investigations on the pharmacological properties of lecithin, a major constituent of oil from the yolk, suggest that it might be involved in cellular metabolism and in repair of membranes (8, 9). Clinical observations have indicated that the topical application of egg yolk oil may be beneficial in relieving pain and promoting wound healing on burn wounds (10). However, there are still relatively limited structured clinical data assessing its specific effectiveness on fractional carbon dioxide laser-induced wounds.

Many studies evaluating the clinical utility of topical agents are retrospective cohorts that are vulnerable to baseline confounding factors and selection bias (11). Reduced differences in the distribution of covariates can be achieved by using statistical methods like propensity score matching and a more objective evaluation basis can be used (12). Besides the clinical efficacy analysis, it is also of clinical interest to anticipate which patients may obtain an optimal therapeutic response. Machine learning algorithms are known to be capable of dealing with complex medical data (13). However, these computational models are difficult to translate clinically due to their lack of interpretability (14). The SHapley Additive exPlanations (SHAP) framework provides a mathematical way to estimate the marginal contribution of each feature, and thus to convert the “black box” prediction into relationships that can be easily understood (15).

The purpose of the present study is to assess the possible clinical impact of using topical egg yolk oil in wound healing and scar remodeling after fractional carbon dioxide laser skin resurfacing. To minimize baseline differences, a propensity score-matched retrospective cohort was created. Using this cohort, we have developed different machine learning algorithms to forecast optimal clinical responses. To help explain the non-linear relationships and possible interactive effects between early recovery outcomes and long-term aesthetic outcomes, subsequent SHAP analysis was performed.

## 2 Materials and methods

### 2.1 Study design and ethical considerations

The study's protocol was approved by the Institutional Review Board of the Affiliated Hospital of Panzhihua University (Approval No. 2024-02-001). The ethics committee waived the need for written informed consent, as this is a retrospective study. All experiments involving human subjects were performed in accordance with the ethical standards of the institutional and national research committees in accordance with the principles of the Declaration of Helsinki (1975) and its later revisions.

### 2.2 Patient selection criteria

A systematic review was conducted on the electronic medical records of patients with atrophic acne scars. Patients with atrophic acne scars, treated with fractional carbon dioxide (CO_2_) laser therapy and aged between 18 and 45 years were selected. The following exclusion criteria were applied: any active skin infections, presence of active inflammatory acne lesions (such as cysts, pustules, or confluent erythema precluding immediate laser resurfacing) within the treatment area, known history of keloid formation, oral isotretinoin treatment in the last 6 months, other scar revision treatments or incomplete clinical and follow-up data. Additionally, to ensure patient safety and minimize confounding, records demonstrating documented egg allergies, immunosuppression, diabetes mellitus, severe coagulation disorders, pregnancy, history of recurrent herpes simplex infections, or recent use of topical retinoids and corticosteroids within one month prior to therapy were strictly excluded. It should be noted that baseline VISIA indices were utilized retrospectively as analytical variables rather than absolute instrumental thresholds for inclusion.

### 2.3 Laser procedure and interventions

The area to be treated was prepared with skin antisepsis using a clear 0.5% chlorhexidine-alcohol solution. To ensure safety and prevent laser interference, the antiseptic was allowed to dry completely prior to procedure commencement, and a topical anaesthetic cream was used. The fractional CO_2_ laser therapy was performed using an ultra-pulsed laser (10,600 nm, DeepFX; Chongqing Jingyu Medical Laser Co., Ltd., Chongqing, China). Strict infection control was maintained by attaching sterile, single-use disposable tips to the laser handpiece for each patient. The fractional handpiece was used with a coverage of 20%–30% and a standardized spot size of 0.12 mm. The pulse duration was consistently maintained at ≤ 5 ms across all patients. The device allows for a range of energies from 40 mJ to 460 mJ, but the energies used in this therapy (usually 50 mJ to 80 mJ) were determined by the severity of each scar and the tolerance of the skin. A non-overlapping single pass scanning technique was used, with the clinical endpoint defined as the appearance of uniform, pinpoint frosting and mild tissue contraction without excessive thermal damage. Laser operators were fully blinded to post-laser topical care protocols during procedure delivery.

The treated area was immediately washed with sterile isotonic saline and dried with sterile gauze. Patients were divided into different groups according to their medical histories for the post-operative procedure. The observation group was topically treated with egg yolk oil (Sichuan Shennong Pharmaceutical Co., Ltd., Sichuan, China; Drug Manufacturing License No. Chuan 20160082, prepared according to the Sichuan Provincial Processing Standards for Chinese Materia Medica, 5 mL/bottle), while the control group was topically treated with physiological saline. It is critical to note that the utilized egg yolk oil is a standardized and sterile pharmaceutical preparation produced in a licensed pharmaceutical manufacturing facility under stringent Good Manufacturing Practice (GMP) protocols, conforming to the microbiological sterility standards of the Chinese Pharmacopoeia (2020 Edition). All patients in the intervention group received the identical standardized formulation from the same commercial manufacturer. Each patient was allocated an individual, factory-sealed bottle to eliminate cross-contamination risks. Pharmacologically, the preparation represents a pure lipid extract enriched with natural phospholipids and lecithin, processed without added fragrances, preservatives, or artificial excipients.

The topical agents were applied twice a day in the designated manner until the wound was completely epithelialized. Operationally, prior to each application, patients gently cleansed the treated area with sterile saline. The egg yolk oil was applied using a direct, open-method technique devoid of occlusive dressings, distributing a thin, uniform layer approximating 0.1 mL per square centimeter across the laser-ablated surface. Apart from the assigned topical agents, both groups received identical basic post-laser wound care, which included gentle cleansing with normal saline and strict sun avoidance measures. The use of supplementary topical antibiotics, corticosteroids, commercial moisturizers, or any other adjunctive scar-revision therapies was strictly prohibited during the initial healing phase to prevent potential confounding of wound healing and pigmentary outcomes. A systematic review of the electronic medical records indicated no occurrences of severe adverse events, such as persistent infections, contact dermatitis, or localized folliculitis, during the immediate post-operative period.

### 2.4 Clinical data extraction

A VISIA Complexion Analysis System was used for clinical assessment and high-resolution photographic documentation was completed using an EOS 400D digital single-lens reflex camera (Canon Inc. Tokyo, Japan). Fifteen clinical indicators were extracted for analysis. Baseline data included age, gender, body mass index (BMI), acne scar duration, baseline Echelle d'Evaluation Clinique des Cicatrices d'Acne (ECCA) score, baseline erythema index and baseline pigmentation index. The parameters of the treatment included laser energy and frequency. Early post-operative parameters were immediate Visual Analogue Scale (VAS) pain score, wound healing time, crusting time and decrusting time. Crusting time was clinically defined as the number of days required for the initial formation of a dry, brownish crust over the entire treated area. Complete decrusting (complete re-epithelialization) was defined as the total detachment of the crusts leaving a continuous, newly epithelialized surface devoid of open wounds or exudate, as evaluated independently by two blinded dermatologists. Follow-up measures included the 1-month erythema index, 1-month pigmentation index, 1-month and 2-month ECCA scores. The main clinical evaluation criterion was the mathematical achievement of an optimal clinical response, which was determined by a 30% or more decrease in the ECCA score from baseline to the 2-month follow up.

### 2.5 Propensity score matching

Baseline confounding factors inherent to retrospective cohorts were considered and a propensity score matching (PSM) procedure was undertaken to reduce the risk of potential selection bias. A multi-variable logistic regression model was developed to estimate the propensity scores using age, gender, BMI, baseline ECCA score, and baseline VISIA scores. Matching was done using a 1:1 nearest neighbor matching algorithm without replacement with a caliper of 0.25 standard deviations of the logit of the propensity score. Although operational variables such as customized laser energy and treatment frequency were not directly embedded in the propensity score calculation model, comprehensive downstream covariate analysis confirmed that these treatment-related parameters achieved an optimal statistical balance between the two cohorts after matching (e.g., *P* = 0.536 for treatment energy, *P* = 0.795 for treatment frequency; detailed in Table 1), thus mitigating their potential confounding effects on final outcomes. This 1:1 nearest-neighbor matching approach with a stringent caliper was selected over alternative propensity score methods (such as inverse probability of treatment weighting or full matching) because it effectively excludes non-comparable historical outlier cases, thereby achieving optimal covariate balance and minimizing sample variance between the two retrospective cohorts in an observational setting (16).

**Table 1:** Baseline demographics and clinical characteristics of the propensity score-matched cohort.

Characteristic	Control Group (*n* = 162)	Egg Yolk Oil Group (*n* = 162)	*P*-value	Statistical Test
Demographics				
Age (Years), Mean ± SD	26.8 ± 4.3	26.5 ± 4.3	0.602	Student's *t*-test
Gender, *n* (%)				
Female	82 (50.6%)	89 (54.9%)		
Male	80 (49.4%)	73 (45.1%)	0.504	Chi-square test
BMI (kg/m ^2^), Mean ± SD	22.4 ± 2.6	22.6 ± 2.7	0.585	Student's *t*-test
Scar Baseline Severity				
Acne Scar Duration (Years), Median [IQR]	5.2 [3.3–7.9]	5.0 [3.0–7.3]	0.359	Wilcoxon rank-sum test
Baseline ECCA Score (Points), Mean ± SD	65.6 ± 14.8	65.1 ± 13.1	0.771	Student's *t*-test
Skin Biophysical Parameters				
Baseline Erythema Index (a.u.), Mean ± SD	388.0 ± 44.1	389.0 ± 46.5	0.834	Student's *t*-test
Baseline Pigmentation Index (a.u.), Mean ± SD	212.8 ± 27.1	213.6 ± 29.8	0.806	Student's *t*-test
Fractional Laser Parameters				
Treatment Energy (mJ), Median [IQR]	66.0 [59.0–72.0]	65.0 [57.0–72.0]	0.536	Wilcoxon rank-sum test
Treatment Frequency (Hz), Mean ± SD	90.2 ± 5.9	90.4 ± 5.6	0.795	Student's *t*-test

### 2.6 Machine learning modeling

Four supervised machine learning algorithms were built using the matched cohort to predict the probability that an optimal clinical response would be achieved: Logistic Regression (LR), Support Vector Machine (SVM), eXtreme Gradient Boosting (XGBoost), and Random Forest (RF). A 5-fold cross-validation technique was used to make the training of the models robust. However, to overcome the imbalance of the binary target, the classes’ weights were adjusted in the algorithmic learning stage, which lessens the effect of misclassification of the minority class and avoids distortion of the decision boundary. A grid search was used for hyperparameter optimization, which was strictly implemented within a nested cross-validation framework (comprising an outer 5-fold cross-validation loop for unbiased performance estimation and an inner 3-fold cross-validation loop for hyperparameter tuning). Within each inner fold, hyperparameters (e.g., number of decision trees and maximum depth in Random Forest) were optimized by minimizing log-loss. Crucially, all reported performance metrics—including ROC-AUC, Precision-Recall curves, calibration curves, and confusion matrices—were derived exclusively from the pooled held-out outer validation folds, thereby avoiding optimistic bias and data leakage.

### 2.7 Model interpretability analysis

In order to make sense of the decision-making logic of the machine learning model that performed best, the SHapley Additive exPlanations (SHAP) framework was used. To estimate the marginal impact of the clinical features on the output of the prediction, SHAP values were calculated. However, it is worth highlighting that the SHAP analysis is only used to show mathematical associations and non-linear dependencies between variables and should not be interpreted as biological causality.

### 2.8 Statistical analysis

The data were processed and statistically analyzed using the R software. Normally distributed continuous data were presented as mea*n* ± standard deviation and analysed using Student's t-test. Continuous variable data that were not normally distributed were reported as median and interquartile range (IQR) and compared using the Wilcoxon rank-sum test. Categorical data were presented in terms of frequency and percentage, and the Chi-square test was used to compare groups. Time-to-event data (wound healing etc.) were analysed by the Kaplan–Meier method and differences between the curves were tested using the log rank test. The Area Under the Receiver Operating Characteristic Curve (AUC) and Precision-Recall curves were used to assess the predictive capabilities of the machine learning models. ROC curves were compared for statistical differences using the DeLong's test. Statistically significant *P* values were set at < 0.05 (two sided).

## 3 Results

### 3.1 Patient selection and baseline characteristics of the propensity score-matched cohort

For this study, a total of 824 electronic medical records were initially screened. Using the pre-defined exclusion criteria (missing clinical files and concurrent administration of other therapies), 539 eligible patients were identified (Figure 1A). There were potential selection biases and baseline confounding factors, inherent in the observational nature of the retrospective data collection. The absolute standardized mean differences (SMD) for the core baseline characteristics (age, gender, initial skin biophysical parameters) were all >0.35 before matching, as shown in the covariate balance plot (Figure 1F). This implies a significant baseline difference between the control and intervention groups, which may affect the possible objective evaluation of the subsequent treatment efficacy.

**Figure 1:**
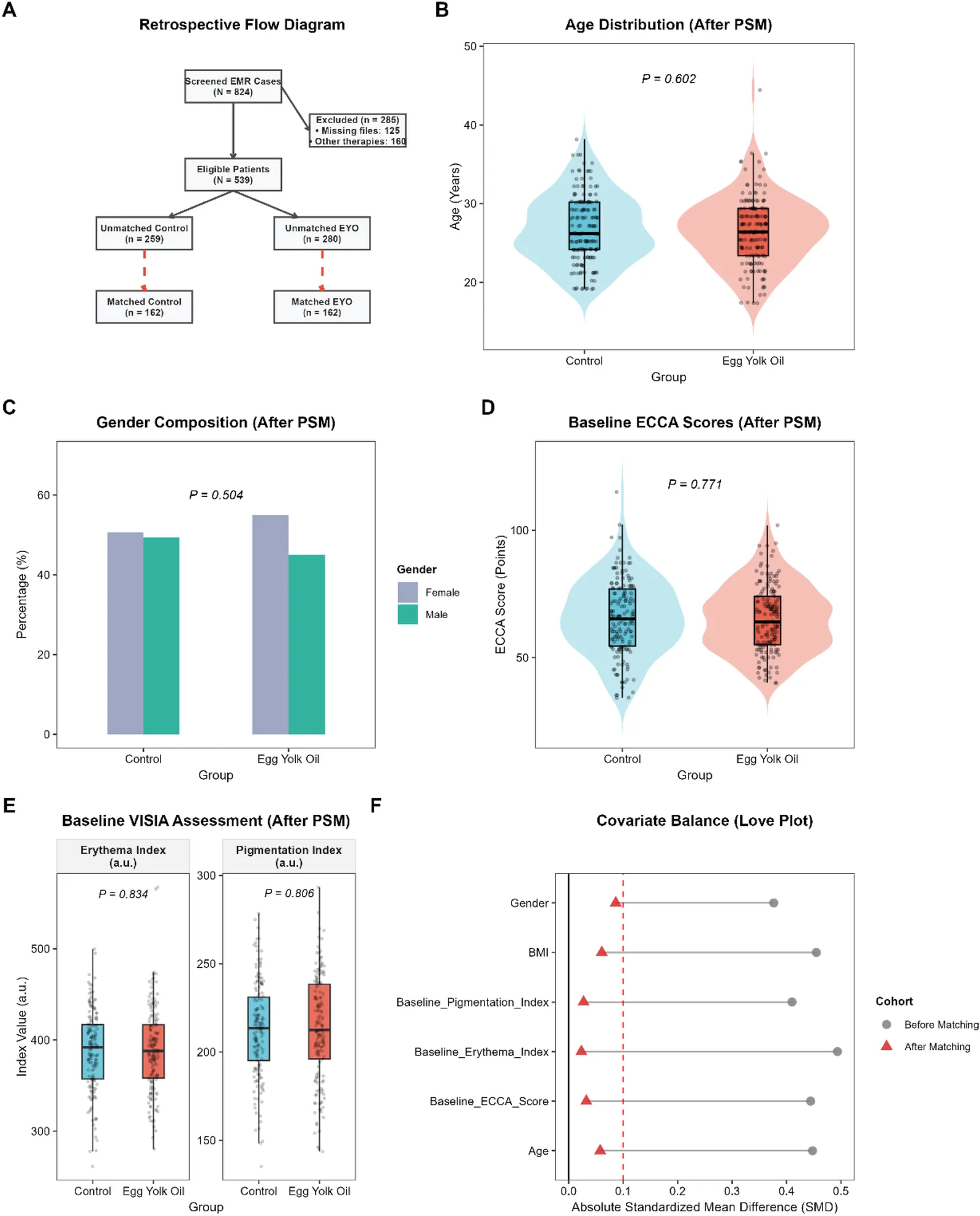
Study flow diagram and baseline characteristics of the propensity score-matched cohort. **(A)** Retrospective flow diagram of patient selection and matching. **(B–E)** Comparisons of age distribution, gender composition, baseline ECCA scores, and baseline VISIA assessments (erythema and pigmentation indices) between the Control and Egg Yolk Oil groups after matching. **(F)** Love plot showing the standardized mean differences (SMD) of covariates before and after propensity score matching.

Propensity score matching (PSM) was used to reduce selection bias and control for these confounding variables, with a 1:1 match completed. Prior to matching, the overlap of propensity score distributions between the two groups was inadequate. During the stringent matching procedure, 215 unmatched patients (118 in the intervention group and 97 in the control group) who fell outside the caliper limits were excluded from the analysis. As a result of this procedure, a final matched cohort of 324 patients was obtained (162 in the control group and 162 in the egg yolk oil group, Figure 1A), showing a highly overlapping and balanced propensity score distribution. After the matching, SMDs of all the covariates included were significantly reduced, becoming below or equal to 0.10 (ranging from 0.02 for erythema index to 0.09 for gender) (Figure 1F and Supplementary Table 1). Detailed baseline characteristics and exact SMDs before and after matching are comprehensively provided in Supplementary Table 1. This reduction suggests that the PSM procedure successfully reduced the initial differences in baseline.

The matched cohort had detailed baseline demographics and clinical characteristics as summarized in Table 1. There were no significant differences between the control and egg yolk oil groups in age (*P* = 0.602, Figure 1B), gender composition (*P* = 0.504, Figure 1C) and BMI (*P* = 0.585). In addition, the scar severities, and skin conditions before treatment were not significantly different between the two groups, with no significant difference in the erythema index (*P* = 0.834; Figure 1E) or pigmentation index (*P* = 0.806; Figure 1E) or the ECCA scores (*P* = 0.771; Figure 1D) before treatment. The groups were also comparable with regard to the treatment parameters (fractional laser energy and frequency) (Table 1). The results of these analyses suggest that the matched cohort offers a balanced starting point (close to a random allocation) for the assessment of wound healing and scar remodeling results.

### 3.2 Early post-operative clinical responses and wound healing dynamics

With a well-balanced baseline cohort, we also assessed early post-operative clinical responses to fractional CO_2_ laser therapy. The objective recovery milestones as indicated by the violin and box plots suggest that EYO might accelerate recovery. In particular, the mean wound healing time of the EYO group was 5.54 days whereas that of the control group was 8.51 days (Figure 2A). This trend was also true for the crusting time (3.03 days vs. 4.48 days; Figure 2B) and the complete decrusting time (7.53 days vs. 10.39 days; Figure 2C). All these differences in time were highly significant (*P*-values < 0.001), suggesting that EYO may have a beneficial effect on early repair of the epidermis and decreasing the inflammatory exudation phase.

**Figure 2:**
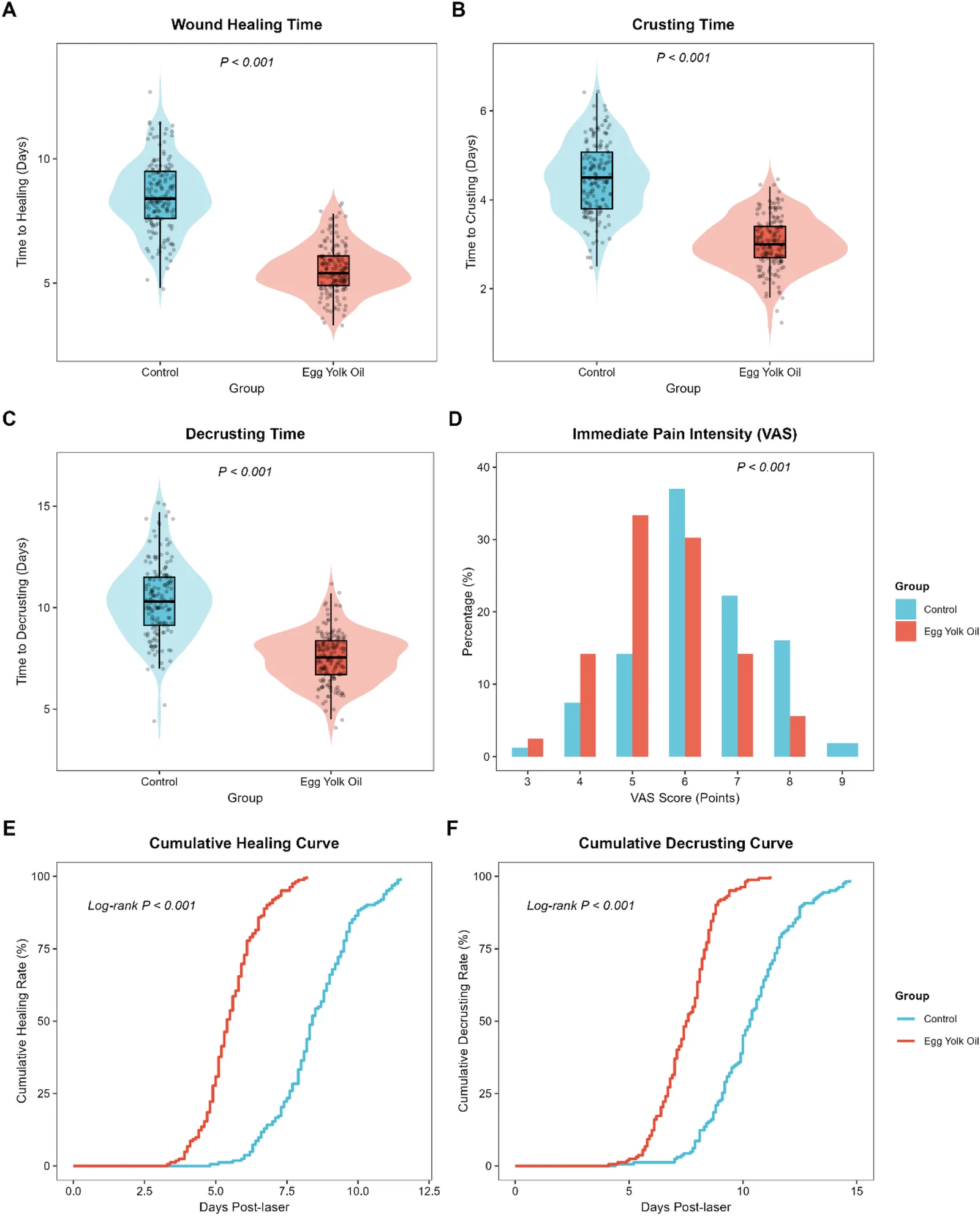
Early post-operative recovery metrics and pain assessment. **(A–C)** Violin and box plots illustrating wound healing time, crusting time, and decrusting time between the two groups. **(D)** Distribution of immediate post-operative pain intensity evaluated by the Visual Analogue Scale (VAS). **(E,F)** Kaplan–Meier curves displaying the cumulative wound healing rate and cumulative decrusting rate over time.

In terms of subjective perception of pain, immediate post-operative Visual Analogue Scale (VAS) scores were analysed. While patient-reported, VAS is a metric whose distribution may be indicative of a soothing effect related to the EYO intervention. A higher proportion of patients in the control group rated their VAS at 6 (37.0%) while a lower proportion was seen in the EYO group (33.3%) as shown in Figure 2D. In addition, higher pain scores (e.g., 7 and 8) were less frequent in the EYO group. This distributional difference was significant (at *P* < 0.001), and may suggest a possible role for EYO in reducing acute post-laser discomfort.

A time-to-event analysis was performed using Kaplan–Meier curves, with complete wound healing and decrusting being sequential clinical endpoints, in order to better reflect the dynamic nature of the wound healing process. This method is helpful in the visualization of the cumulative probability of recovery over the follow-up days. The cumulative healing curve (Figure 2E) and the cumulative decrusting curve (Figure 2F) show a definite leftward shift of the EYO group. Log-rank tests (both *P* < 0.001) also suggest that patients receiving topical EYO were much more likely to achieve these early recovery milestones than patients in the control group.

### 3.3 Medium-term follow-up evaluations and scar remodeling outcomes

We noted the early acceleration of epidermal repair following application of topical egg yolk oil (EYO) and decided to expand our evaluation to medium-term (1 month and 2 months) aesthetic and functional recoveries. Dynamic monitoring of skin biophysical parameters showed a steady reduction in erythema and pigmentation after laser in both groups. Interestingly, a greater decline was observed in the EYO group. One month after the intervention, the EYO group had a mean erythema index of 304.88 a.u. while the control group had a mean erythema index of 328.62 a.u. (*P* < 0.001; Figure 3A). Likewise, the mean pigmentation index (PI) was also significantly lower in the EYO group compared to the control group (171.15 a.u. vs. 196.31 a.u., *P* < 0.001; Figure 3B), suggesting that EYO may have the potential to reduce the risk of post-inflammatory hyperpigmentation (PIH) and persistent erythema.

**Figure 3:**
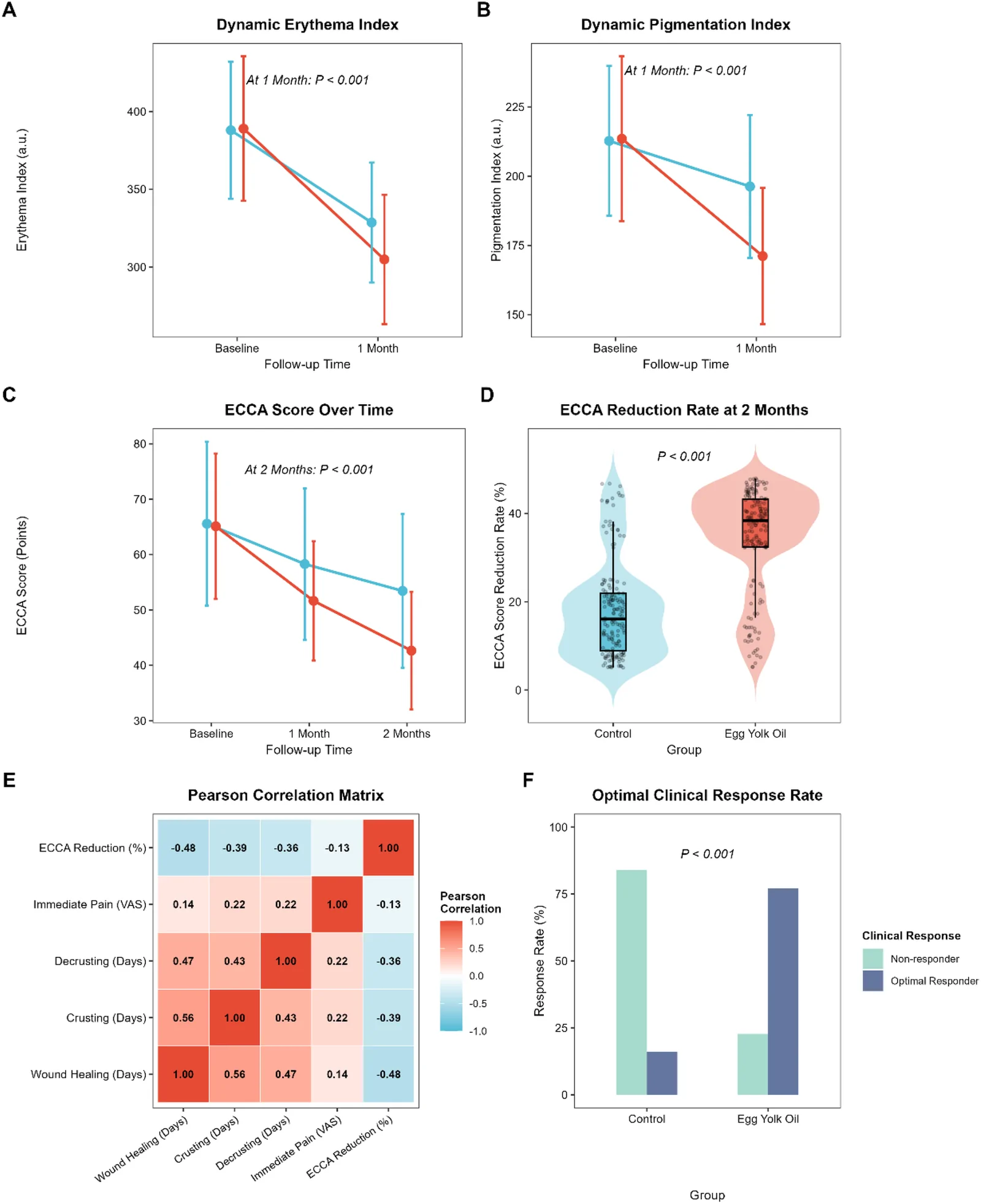
Follow-up clinical evaluations and correlation analysis. **(A–C)** Dynamic changes in the erythema index, pigmentation index, and ECCA scores over the follow-up periods. **(D)** ECCA score reduction rate at 2 months post-treatment. **(E)** Pearson correlation matrix exploring the potential associations among early recovery indicators and final ECCA reduction. **(F)** Comparison of the optimal clinical response rate (defined as an ECCA reduction ≥ 30%) between the two groups.

The ECCA (a clinical acne scar grading) score was serially evaluated from baseline to the 2-month endpoint. The ECCA score for both groups decreased over time but the EYO group decreased at a much higher rate as shown in Figure 3C. The score reduction rate of the ECCA in the EYO group (mean reduction 34.16%) was significantly higher than that of the control group (18.35%) after 2 months (*P* < 0.001, Figure 3D). This suggests that the immediate wound healing properties of EYO could result in better long-term scar remodeling.

To explore temporal relationships of early recovery measures with ultimate clinical outcomes further, a Pearson correlation matrix was included (Figure 3E). The results suggested that there was a negative correlation between the final ECCA reduction rate and wound healing time (r = −0.48), crusting time (r = −0.39) and decrusting time (r = −0.36). This inverse relationship was consistent with the clinical intuition that a longer inflammatory period and a slower epithelialization might be detrimental to later dermal collagen reorganization.

Finally, patients were categorized by their best clinical response, considered as a decrease in ECCA score of ≥30%. The difference between the two groups in terms of categorical comparison was notable; 125 of 162 in EYO group (77.16%) were optimal responders as compared to only 26 in control group (16.05%) (*P* < 0.001; Figure 3F). These medium-term assessments taken together suggest that topical EYO may not only increase the speed of acute wound healing, but also might optimize the effectiveness of fractional CO_2_ laser therapy on atrophic acne scars.

### 3.4 Performance evaluation and model validation

To explore the potential of machine learning algorithms to predict patients who would be likely to achieve optimal clinical responses, four different models were developed and validated using the matched cohort data. The Random Forest model had the best AUC of 0.889, while XGBoost, SVM and Logistic Regression followed in order with AUCs of 0.834, 0.801 and 0.767 respectively, as shown in the ROC curves (Figure 4A). The DeLong's test to compare the performance of the Random Forest model and the Logistic Regression model provided a Z-statistic of 5.84 with a *P*-value < 0.001, suggesting that ensemble-based machine learning methods may have potential advantages over traditional regression models in quantifying the non-linear complexities of clinical data. This was also corroborated by the Precision-Recall curves (Figure 4B) as Random Forest had the highest precision value across most recall values.

**Figure 4:**
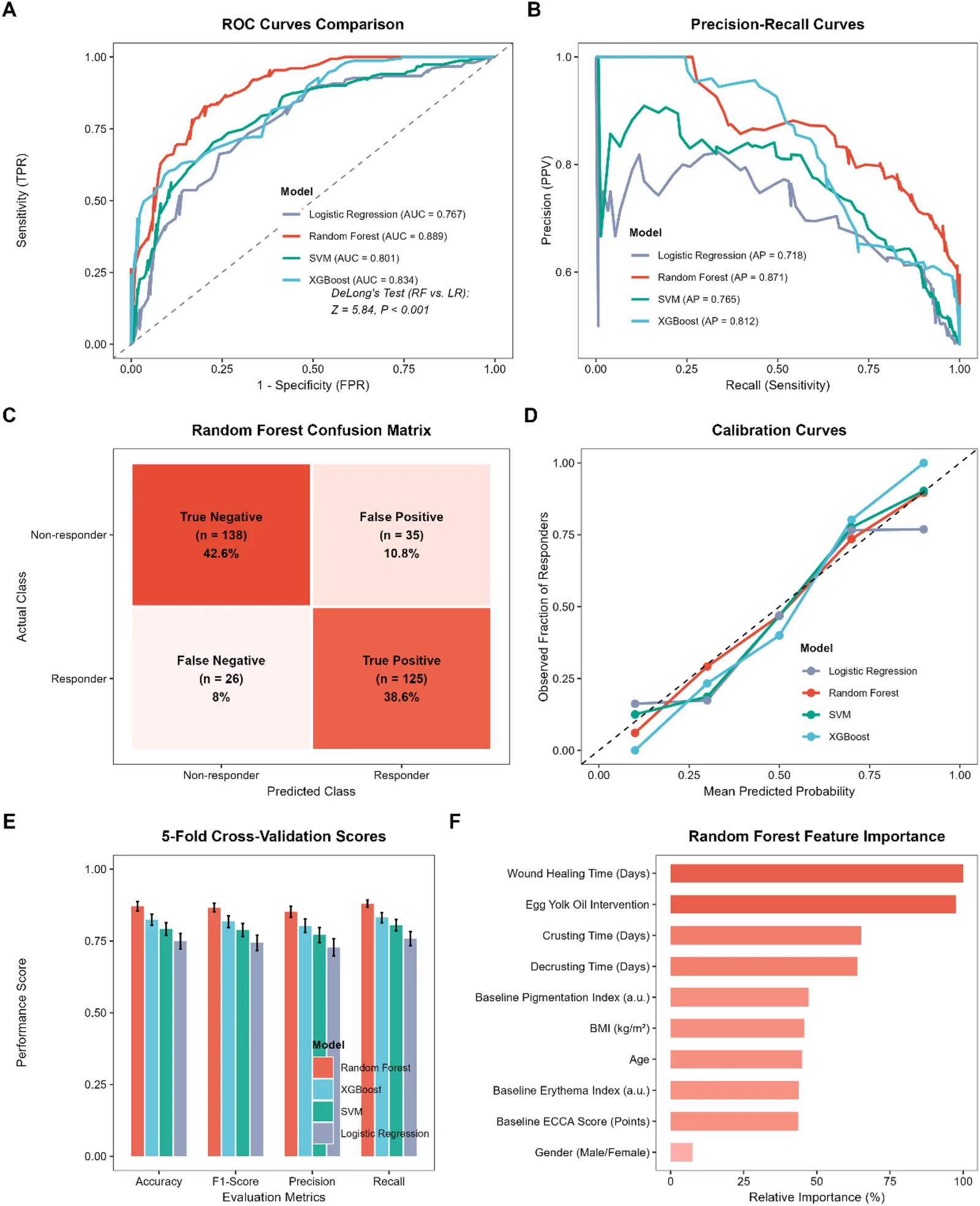
Performance evaluation of different machine learning models for predicting optimal clinical response. **(A)** Receiver Operating Characteristic (ROC) curves and corresponding Area Under the Curve (AUC) values. **(B)** Precision-Recall (PR) curves. **(C)** Confusion matrix of the Random Forest model. **(D)** Calibration curves comparing the predicted probabilities with observed fractions of responders. **(E)** 5-fold cross-validation scores for accuracy, F1-score, precision, and recall across the evaluated models. **(F)** Relative feature importance derived from the Random Forest model.

The performance of the Random Forest model was further assessed with a confusion matrix (Figure 4C) showing a high classification accuracy with 138 true negatives and 125 true positives out of 324 patients. The calibration curve (Figure 4D) showed good calibration, with the predicted probabilities from the Random Forest model being similar to the observed fractions of responders. To assess the stability of the model, 5-fold cross validation (CV) was performed (Figure 4E). In line with the main assessment parameters, the Random Forest model consistently performed well in terms of accuracy, F1-score, precision, and recall, suggesting that this model has the potential to be used as a reliable tool for prognostic stratification.

Finally, to shed light on the clinical relevance of the model, we analysed feature importance using the Random Forest algorithm (Figure 4F). The results suggest that the two variables “Wound Healing Time” and “Egg Yolk Oil Intervention” had the highest relative importance and hence, were the main contributors to the predictive accuracy of the model. Baseline demographic factors such as gender had a relatively lower impact, whereas early recovery metrics like “Crusting Time” and “Decrusting Time” showed moderate importance. This ranking of feature importance is in line with our earlier clinical findings that the dynamics during the early post-operative period are potentially crucial factors in the long-term therapeutic impact of fractional CO_2_ laser treatments.

### 3.5 Model interpretability and SHAP interaction analysis

The Random Forest model showed good predictive ability but it is important to understand the underlying relationships that drive these predictions to be clinically useful. To help explain the reasoning behind the model's prediction, we adopted a game-theoretic method called SHapley Additive exPlanations (SHAP) to quantify the contribution of each feature to the prediction, although it represents an association rather than causality.

The SHAP summary beeswarm plot (Figure 5A) shows the impacts of features across the entire cohort. It shows that higher feature values (red dots) of “Egg Yolk Oil Intervention” correspond to higher SHAP values, which suggests a positive correlation between the two, that is, a good link with optimal clinical responses. On the other hand, a longer “Wound Healing Time” (red dots) is mostly associated with negative SHAP values suggesting that this attribute may have a negative impact on the predicted outcome. These contributions are also quantitatively evaluated by the absolute SHAP values (Figure 5B) and were ranked accordingly, with the contributions of the intervention strategy and early wound healing dynamics being the most influential factors in the model.

**Figure 5:**
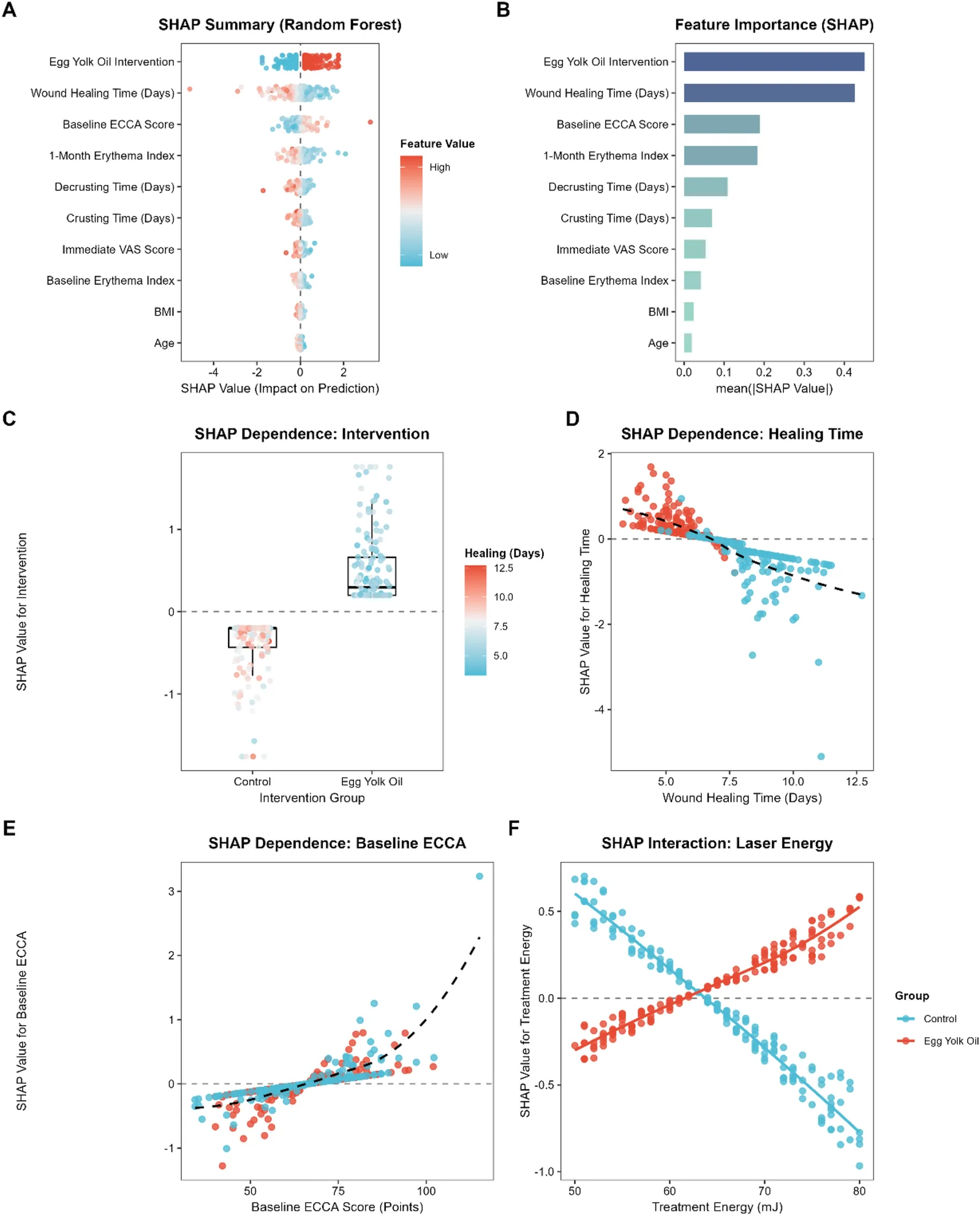
SHapley additive exPlanations (SHAP) analysis for the random forest model. **(A)** SHAP summary beeswarm plot illustrating the distribution of SHAP values for each feature. **(B)** Mean absolute SHAP values representing overall feature importance. **(C–E)** SHAP dependence plots for the Egg Yolk Oil intervention, wound healing time, and baseline ECCA score, suggesting their non-linear marginal effects on the model's output. **(F)** SHAP interaction plot indicating the potential interactive effect between treatment energy and intervention groups on the prediction.

To study the interplay between individual features and predictions, we created SHAP dependence plots. The SHAP values of all the patients in the egg yolk oil intervention group, as shown in Figure 5C, demonstrate a positive shift, while the ones in the control group show a negative shift. Notably, there is a color overlay which suggests there might be some interaction with the wound healing time, with the intervention group (blue dots) showing shorter times. The relationship between wound healing time and the predictions is found to be non-linear from the dependence plot (Figure 5D). SHAP values are relatively positive or neutral for the first 6–7 days of healing times, but show a dramatic drop after that. This suggests the possibility that there may be a timepoint after which delayed re-epithelialisation may have a negative correlation with long term scar improvement. By the same token, the dependence plot for baseline ECCA scores (Figure 5E) shows an increase in the SHAP value as the severity of the acne scar increases, which may suggest that patients with higher (more severe) baseline ECCA scores may have a proportionately greater potential for relative improvement.

In addition, we explored the possible interaction between the treatment intensity and the post-operative care with a SHAP interaction plot (Figure 5F). The analysis shows a divergent pattern by the intervention group. As laser treatment energy is increased, the respective SHAP values in the control group move increasingly to the negative side, and this could represent a risk for greater thermal damage when there is no protection of the epidermis. For the egg yolk oil group, on the other hand, there is an upward trend in the SHAP value with increasing treatment energies. This divergent tendency suggests that there may be a potential statistical association suggesting a synergistic relationship between the use of egg yolk oil and the modulation of increased energy settings, which could lead to a better therapeutic effect. It is essential to reiterate that these SHAP interactions reflect mathematical dependencies within the model rather than confirmed biological causality.

## 4 Discussion

Fractional carbon dioxide lasers continue to be a major technique for treating atrophic acne scarring, but the treatment of thermal damage and extended epidermal healing times are still a clinical challenge (17). In this retrospective propensity score matched study, we observed that the topical use of egg yolk oil suggests an association with accelerated early wound healing and that it demonstrates a potential link to better medium-term scar remodeling, which was also supported by machine learning and SHAP interpretability analysis.

Past publications have indicated that it is essential to provide a moist and lipid-rich environment in the post ablative laser therapy epidermal resurfacing process (18, 19). These concepts are echoed by our clinical data showing that the egg yolk oil group had faster complete decrusting as well as less immediate pain intensity. Moreover, the decrease of both erythema and pigmentation indices during the dynamic period suggests that egg yolk oil may be useful to regulate early inflammatory responses. Studies on the traditional use for burns have suggested that a major compound of egg yolk oil, lecithin, may be involved in cell membrane repair (20). The faster wound closure seen in our cohort could in part account for the decrease in post-inflammatory hyperpigmentation (PIH), potentially leading to better aesthetic results at the two-month follow-up seen in our results.

A major criticism of retrospective cohort studies is that they are vulnerable to a tendency to selection bias and baseline confounding (21). This methodological concern has been addressed by employing a robust propensity score matching mechanism in our study (22). As shown in the covariate balance analysis, the matching was effective in reducing the differences in the demographic and clinical features (including the initial scar severity and laser energy parameters). This statistical adjustment gives a more accurate platform from which to estimate the intervention effect, and suggests that the clinical improvements seen are more likely to be due to the topical intervention and not because of underlying differences among patients.

Machine learning algorithms were incorporated to further examine the predictive factors for optimal clinical response. Random Forest showed good discriminative capability as compared to the conventional regression methods. More importantly, the following SHAP analysis gave interpretable insights into the contribution of each feature, which are associations rather than causal relationships (23). SHAP dependence plots showed a non-linear relationship with regard to wound healing time, suggesting that after around 7 days of healing, the probability of optimal scar remodeling sharply decreased. Furthermore, the SHAP interaction analysis revealed a negative interaction between treatment energy and the intervention. The use of egg yolk oil seemed to ensure positive predictive contribution at higher laser energies, while higher laser energies in the control group were found to have negative predictive contribution. Based strictly on these statistical associations, we cautiously speculate that the topical intervention might potentially associate with a protective barrier effect, which might increase the safe therapeutic window of ablative laser interventions.

This study has several major limitations. First, this is a retrospective study conducted at a single center, meaning the patient population and laser therapy protocols might not be universally generalizable; multicenter prospective trials are needed to validate these findings. Second, the follow-up period was relatively short (two months post-treatment), which might not adequately capture the long-term collagen remodeling process and the late onset of potential complications associated with atrophic acne scars. Since neocollagenesis following ablative laser resurfacing typically continues to evolve for six months or longer, the improvements observed at two months should be cautiously interpreted as early-to-mid-term aesthetic enhancements rather than the final, definitive clinical outcomes. Third, the control group utilized physiological saline rather than a standardized lipid-based vehicle comparator. Consequently, it remains challenging to definitively differentiate the specific pharmacological benefits of egg yolk-derived constituents (such as lecithin) from the generic protective, occlusive, and moisture-retaining effects of a lipid-rich barrier. Future studies should incorporate appropriate vehicle controls to isolate these active properties. Fourth, the machine learning models were developed and evaluated internally using cross-validation without an independent, external hold-out test cohort. Consequently, the predictive performance metrics should be interpreted with caution, and the generalizability of these algorithms to distinct clinical populations remains to be further assessed.

## 5 Conclusion

In conclusion, this retrospective study using propensity score matching suggests that topical use of egg yolk oil after fractional CO_2_ laser treatment may facilitate early epidermal healing and minimize discomfort after treatment in patients with atrophic acne scars. In addition, mid-term follow-up assessments suggest that this intervention is linked to improved scar remodeling, and possible decreases in post-inflammatory erythema and hyper-pigmentation. The use of machine learning models, especially Random Forest in combination with SHAP analysis, also suggests that early wound closure and optimal laser energy modulation are key factors related to clinical efficacy. While this study has inherent limitations due to the retrospective nature and only a single center, it offers reasonably good clinical evidence suggesting an association between topical egg yolk oil application and improved post-laser wound recovery. Longer duration follow-up multicenter prospective studies are needed to further validate these findings.

## Data Availability

The raw data supporting the conclusions of this article will be made available by the authors, without undue reservation.
